# Modeling the Effect of Intra-Voxel Diffusion of Contrast Agent on the Quantitative Analysis of Dynamic Contrast Enhanced Magnetic Resonance Imaging

**DOI:** 10.1371/journal.pone.0108726

**Published:** 2014-10-02

**Authors:** Stephanie L. Barnes, C. Chad Quarles, Thomas E. Yankeelov

**Affiliations:** 1 Vanderbilt University Institute of Imaging Science, Vanderbilt University, Nashville, Tennessee, United States of America; 2 Department of Radiology and Radiological Sciences, Vanderbilt University, Nashville, Tennessee, United States of America; 3 Department of Biomedical Engineering, Vanderbilt University, Nashville, Tennessee, United States of America; 4 Department of Physics and Astronomy, Vanderbilt University, Nashville, Tennessee, United States of America; 5 Department of Cancer Biology, Vanderbilt University, Nashville, Tennessee, United States of America; 6 Vanderbilt-Ingram Cancer Center, Vanderbilt University, Nashville, Tennessee, United States of America; 7 Program in Chemical and Physical Biology, Vanderbilt University, Nashville, Tennessee, United States of America; Institute for Frontier Medical Sciences, Kyoto University, Japan

## Abstract

Quantitative dynamic contrast enhanced magnetic resonance imaging (DCE-MRI) provides estimates of physiologically relevant parameters related to tissue blood flow, vascular permeability, and tissue volume fractions which can then be used for prognostic and diagnostic reasons. However, standard techniques for DCE-MRI analysis ignore intra-voxel diffusion, which may play an important role in contrast agent distribution and voxel signal intensity and, thus, will affect quantification of the aforementioned parameters. To investigate the effect of intra-voxel diffusion on quantitative DCE-MRI, we developed a finite element model of contrast enhancement at the voxel level. For diffusion in the range of that expected for gadolinium chelates in tissue (i.e., 1×10^−4^ to 4×10^−4^ mm^2^/s), parameterization errors range from −58% to 12% for *K^trans^*, −9% to 8% for *v_e_*, and −60% to 213% for *v_p_* over the range of *K^trans^*, *v_e_*, *v_p_*, and temporal resolutions investigated. Thus the results show that diffusion has a significant effect on parameterization using standard techniques.

## Introduction

Dynamic contrast enhanced magnetic resonance imaging (DCE-MRI) involves the serial acquisition of images before, during, and after the injection of a paramagnetic contrast agent into a peripheral vein. As the contrast agent is exchanged between the vascular space and the extravascular extracellular space (EES), it interacts with the surrounding tissue water molecules, shortening the tissue's native *T_1_* relaxation time and resulting in a quantifiable increase in the signal intensity (*SI*) on a *T_1_*-weighted sequence. By considering the time series data, a *SI* time course is formed which can be analyzed with an appropriate pharmacokinetic model to estimate biologically relevant parameters describing, for example, tissue blood flow, vessel permeability, and tissue volume fractions.

The pharmacokinetic models most commonly used to describe the contrast agent kinetics within a tissue were adapted from the model developed by Kety which described the exchange of an inert gas between two compartments within a tissue [1]. These “standard models”, as applied to DCE-MRI, account for active delivery of the contrast agent via the vasculature and exchange of the contrast agent between the vascular space and the EES [2], [3], [4]. Generally speaking, the models used in DCE-MRI analysis neglect any diffusion of the contrast agent that may occur within the tissue between well and poorly vascularized areas. The effect of contrast agent diffusion may not be trivial in pathologic conditions where spatial heterogeneity of the vasculature is routinely observed, as is the case, for example, in tumors [5]. Thus, in tissues in which diffusion of the contrast agent contributes substantially to the observed dynamic signal enhancement, it is possible that the established models – which are not designed to account for diffusion of contrast agent – may estimate pharmacokinetic parameter values with reduced reliability. As these are the same model parameters that have been shown to assist in both diagnosis [6], [7], [8], [9], [10] and prognosis [11], [12], [13], [14], [15], [16], [17], [18], [19], [20], it is of great import to accurately (and precisely) assign their values. Previous studies have hypothesized that diffusion of contrast agent within the tissue of interest may introduce errors when utilizing the standard models for analyzing DCE-MRI data [21], [22], [23], [24], [25].

Though the potential for contrast agent diffusion effects may be recognized, literature investigating the effect of diffusion is limited. Pellerin *et al*. used a finite difference model to study the effect of diffusion in DCE [21]. The work presented a diffusion-perfusion (DP) model which incorporated voxel to voxel diffusion into the standard model. Using assumed diffusion coefficients from the measurement of the water apparent diffusion coefficient (ADC), model optimization resulted in assignment of *K^trans^* and *v_e_* for each voxel. The work showed a quantitative improvement in the parameter assignment on a voxel basis using the DP model as compared to the standard model both in simulated cases where a distinct delineation between well and poorly perfused regions existed, and in a xenograft tumor which showed evidence of diffusion in which unphysiological values of *v_e_* were assigned by the standard model. Fluckiger *et al.* further analyzed this situation, modifying the DP model to make the voxel diffusion coefficients independent of the other voxels, yielding a more computationally tractable model; they termed this model the diffusion compensated Tofts-Kety model (DTK) [22]. With this model, the authors were able to show an increase in accuracy of parameter assignment over the standard model, both quantitatively in simulated data, and qualitatively in preclinical experimental data. Jia *et al.* calculated a contrast agent diffusion coefficient (CDC) in colorectal liver metastases [23]. To visually assess the effect of diffusion, the authors applied an onion-peeling algorithm to generate pixel-wide layers within the lesion, and then visualized the *SI* curves of each layer. The shape of the curve during the extravascular phase demonstrated the effect of diffusion on the contrast agent concentration within the lesion. The CDC was quantified by evaluating the rate of gradient decrease in the signal intensity within the region as described by a monoexponetial decay. Fitting the decay equation to the imaging data resulted in a decay factor, which, through a defined relationship, was used to calculate the CDC. The authors found the CDC to be a repeatable value that described the heterogeneity of the lesions.

More recently, Sourbron [24] has proposed a field theory for tracer-kinetic studies in medical imaging. In this work, the author develops a more general framework that employs the specific structure of the data available from dynamic imaging studies. In particular, the relevant (desired) tissue parameters are functions of space which can be measured by analyzing the temporal and spatial patterns in the time dependent concentration time courses. The theory allows for the rigorous examination of the effects of convective or diffusive exchange between voxels.

It is important to note that all of the above efforts focused on *inter*-voxel diffusion of contrast agent; the literature focusing on intra-voxel diffusion is even more limited. Pannetier *et al*. performed an investigation of the effect of intra-voxel contrast agent diffusion on magnetic field perturbations and susceptibility and the parameterization error of *k_ep_* (≡*K^trans^*/*v_e_*) and blood volume fraction (BVF) [25]. The authors found that contrast agent diffusion did have an effect on both permeability estimates and the plasma tissue fraction. Additionally, both parameters were also strongly influenced by scan parameters, particularly the echo time. However, the authors' investigation did not focus on the inclusion of cells in the extravascular space, which can be highly variable in various tissue pathologies and would presumably further emphasize the effect of contrast agent diffusion, thus making it an important point of consideration.

The aim of the present effort is to evaluate the propensity of *intra*-voxel diffusion to affect the parameterization of the tissue parameters *via* the extended (Tofts) models. To do so, we generated a finite element model (FEM) of a representative tissue domain that utilized both active delivery to the voxel, which was represented by means similar to the standard model, and passive diffusion within the voxel, which was represented by the classical diffusion equation. The FEM generated a tissue concentration distribution which could then be utilized to calculate a dynamic tissue *SI* time course. The tissue's dynamic *SI* was evaluated at various values of contrast agent diffusion to assess the effect of intra-voxel diffusion on the measured *SI* within the voxel. Given the assumption of well-mixed compartments of the common DCE-MRI models, we hypothesize that at high coefficients of diffusion (which allow sufficient mobility of the contrast agent) these methods will accurately model the domain. We further hypothesize that error in pharmacokinetic parameter estimates will increase with decreasing coefficients of diffusion, as the assumption of a well-mixed domain becomes invalid. This contribution investigates the effect of varying distributions of the tissue volume fractions, specifically including cellular and vascular structures, and provides a systematic characterization of the effect of diffusion on the accuracy of the frequently used DCE-MRI modeling approaches.

## Methods

### Theory

The most commonly used approach for quantitative analysis of DCE-MRI data is the so-called standard model which utilizes a two-compartment model to describe the concentration change of the contrast agent within the tissue: 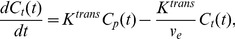
(1)where *C_t_*(*t*) and *C_p_*(*t*) are time courses of the concentration of contrast agent in the tissue and blood plasma (the arterial input function, or AIF), respectively, *K^trans^* is the volume transfer constant, and *v_e_* is the extravascular extracellular volume fraction [26]. The standard model only considers the contrast agent in the EES and neglects the portion of tissue that is composed of vasculature. However, in some cases, the plasma fraction of the tissue is not insignificant and may introduce error into the parameterization of the standard model [27], [28]. Thus, investigators have amended the standard model to include the contribution of the plasma space within the tissue:
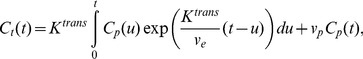
(2)where *v_p_* is the vascular volume fraction within the section of tissue under investigation. With measured *C_p_*(*t*) and *C_t_*(*t*) time-courses, either the standard model or the extended model can be fit to the data to estimate the parameters *K^trans^*, *v_e_*, and, in the case of the extended model, *v_p_*.

In order to investigate the effect of intra-voxel contrast agent diffusion on the ability to accurately estimate DCE parameters using the extended model, we developed a two-dimensional finite element model (FEM) describing perfusion and diffusion within a tissue domain. The FEM was developed using the Galerkin approach [29] with the standard Lagrange polynomial interpolants [30], and a Crank-Nicholson [31] scheme for the time domain. While details of this approach are provided elsewhere [29], [30], [31], we now discuss the salient features for the sake of clarity in our particular application.

In FEM analysis, the solution, *C*(*x,y*) (which, in this work, is the contrast agent concentration at the nodal indices), is approximated by a coefficient expansion using a set of basis functions:
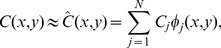
(3)where 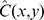 is the approximate solution, *C_j_* are the unknown coefficients, and 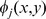 are the spatially dependent basis functions. Additionally, the weighted residual method requires that the residual error between the approximate and analytical solutions vanishes over a weighted integral, and thus a set of weighting functions is required:
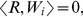
(4)where *R* is the resultant residual of the approximation of *C(x,y)*, *W_i_* (*i*  =  1:*N*) is the set of *N* weighting functions, and 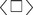 indicates integration over the problem domain. Selection of the basis functions and weighting functions allows for the determination of the unknown coefficients, *C_j_*; in the Galerkin method, the weighting functions and the basis functions are the same. The Lagrange polynomial is commonly utilized as the basis and weighting functions; this family of polynomials is *C^0^* continuous, and the *i^th^* polynomial,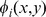, is defined such that the polynomial is equal to unity at node *i* and is 0 at all other nodes. Thus, by definition, the unknown coefficients become the desired nodal solutions.

To address the time stepping of the solution, a Crank-Nicolson approach was used. Temporal integration can be approximated by: 
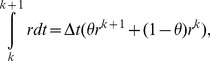
(5)where *r* is the integrand, *k* and *k*+1 are the two time steps, *θ* is a weight, and *Δt* is the time between sequential steps (i.e., *k* and *k*+1). When *θ* is assigned a value of 0, the standard forward Euler approximation to integration is achieved, while a value of 1 yields the standard backward Euler approximation. Assigning *θ* a value of 0.5 results in the trapezoidal integration approximation, or what is also referred to as the Crank-Nicolson approach. The Crank-Nicolson scheme is an implicit temporal differencing method which, with *θ*  =  0.5, assigns equal weighting to the previous and current steps. The advantage to this approach is the increased temporal accuracy relative to the other approaches coupled with the unconditional stability of the dynamic system [29].

**Figure 1:**
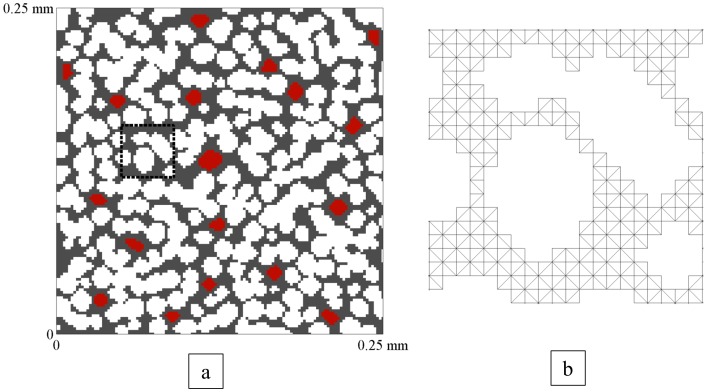
A Representative Voxel Domain. a.) The simulated compartmental domain. The gray area represents *v_e_*, the white voids represent cells (1-*v_e_*-v_p_), and the red spaces indicate vessels (*v_p_*). This domain has a *v_e_* of 0.39 and a *v_p_* of 0.03. Each side of the voxel is 250 micrometers. b.) An example of the triangular mesh (corresponding to the black dashed line in panel a) which fills *v_e_*.

For this work, the domain of interest was a square (i.e., a 2D voxel) with one (or multiple) null region(s), representing the perfusing region(s) (i.e., the vascular space, *v_p_*), and multiple elliptical voids representing packed cells (i.e., the extravascular intracellular space, 1-*v_e_*-*v_p_*) into which the contrast agent could not diffuse (Fig. 1). The remainder of the domain consisted of the EES (*v_e_*) into which the contrast agent could diffuse. The FEM utilized the standard diffusion equation throughout the domain to describe the contrast agent diffusion within the tissue:
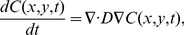
(6)where *D* is the coefficient of diffusion for the contrast agent and *C*(*x,y,t*) is the dynamic, spatially dependent contrast agent concentration. Eq. (6) is only applied within the *v_e_* space of the tissue, so, when the entire domain is considered, *C* in the general diffusion equation becomes *C_e_* relative to the extended Tofts model.

The vessel boundaries, and hence the input of contrast agent into the domain, were handled through the boundary integral that can be introduced into the Galerkin weak form of the equation by means of the second order differential. The Galerkin weak form of the equation (before expanding *C*) is:
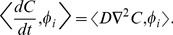
(7)


This second order differential can be integrated by parts, and the boundary integral introduced *via* the divergence theorem:
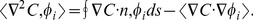
(8)


Substituting (8) into (7) gives an alternate weak form of the diffusion equation; in this format, the appearance of the boundary integral allows for consideration of the movement of the contrast agent across the closed boundaries of the domain. At the cell boundaries and the external boundaries of the extended domain (discussed below), the integral was set equal to 0 to dictate no flux of the contrast agent at those boundaries. However, at the vessel boundaries, flux will occur, and the normal derivative at the boundary can be described by the difference in the concentration between the plasma and the tissue domain, and a transfer coefficient (similar to the standard equation above), as:
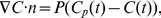
(9)where *P* describes the transfer coefficient between the plasma and the tissue and *C_p_* is as previously defined. *P* is related to *K^trans^* by:
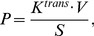
(10)where *S* is the surface area of the vessels and *V* is the volume of the voxel [32]. Since the simulation is presented in 2D, *S* is the length of the vessel boundaries and *V* is the area of the voxel. This relation was chosen so that at high *D* (i.e., *D*>10^−3^ mm^2^/s), the amount of contrast agent in the EES was the same as that predicted by the standard model. In generating the FEM, the condition defined in (9) was substituted for 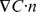 at the boundaries of the perfusing regions (i.e., around all vessels within the domain) to provide the flux of the contrast agent at the vessel boundaries. Thus, the simulations consisted of introduction of the contrast agent to the system at the vessel boundaries *via* (8), and then diffusion throughout the appropriate regions of the domain as described by (6). This system simulated the three compartment (*v_e_*, *v_eis_*, and *v_p_*) model described by (2) while allowing the additional consideration of contrast agent distribution, due to diffusion away from the delivery regions, in the voxel domain.

### Simulations

A representative voxel domain utilized in these simulations is shown in Fig. 1. The domains were generated by selecting an appropriate two-dimensional slice from a three-dimensional structure containing packed ellipsoids generated utilizing an ellipsoid packing algorithm [33], [34]. Each domain consists of three components: the extravascular intracellular space (EIS, 1-*v_e_*-*v_p_*), which is impermeable to the contrast agent, the EES (*v_e_*), into which the contrast agent will diffuse, and the vascular space (*v_p_*) which provides the delivery of the contrast agent to the domain. In Fig. 1, the EIS is represented by the white ellipsoids, the EES is the gray region, and the vascular space is denoted by the red regions. Thus, the volume fractions typically identified in quantitative DCE-MRI analysis were physically defined within the domain. In generating the mesh of the system, only the EES was meshed (i.e., filled with triangular elements) while the EIS and vascular spaces were left as voids. This is appropriate for the case in which the contrast agent cannot enter the EIS (as is true for the common gadolinium chelates frequently employed in DCE-MRI), and thus all that is required is an impermeable boundary at the borders of the cells. Additionally, the concentration in the plasma space is considered uniformly defined by the AIF, and therefore again the interaction at the boundary between the vascular space and the EES is the only area of concern in regard to delivery of the contrast agent to the system.

The domain size utilized in this work was selected to be a region 250 µm on a side, which is a common voxel size for a preclinical study. The average edge length of the triangular elements in the domain mesh is 2 µm. The time step (*Δt*, Eq. (11)) was calculated for each simulation based on mesh parameters and *D*, and was checked to ensure that the simulation was discretized in a manner that specifically retained the peak and other pertinent features of the AIF. Simulations were run for various combinations of *K^trans^*, *v_e_*, and *v_p_* in order to analyze the effect of each on parameterization error. The *K^trans^*, *v_e_* and *v_p_* values were [0.1 0.4 0.7] min^−1^, [0.25 0.39 0.55] and [0.01 0.03 0.06], respectively. While changes in *K^trans^* were implemented by simply varying the values input into the simulation, changes in *v_e_* and *v_p_* required changes in the voxel domain itself. Increasing or decreasing *v_e_* meant removing or adding cells, respectively, while increasing or decreasing *v_p_* meant increasing or decreasing, respectively, the number of vessels in the voxel domain. The average cell diameter utilized in the domains was approximately 14 µm and the average vessel diameter was approximately 8 µm. For the simulations, an extended domain, which was larger than the voxel of interest, was generated in order to allow for boundary conditions at the edges of the voxel of interest that were realistically defined. Specifically, the (250 µm)^2^ voxel was surrounded by eight other identical (250 µm)^2^ voxels, yielding an extended domain size of (750 µm)^2^. When the values for voxel contrast agent concentration and voxel *SI* were calculated, they were calculated only for the central (250 µm)^2^ voxel of interest. In this way, artificial boundary conditions were not assigned at the edge of the voxel, but rather contrast agent was allowed to diffuse between voxels, as would happen physiologically.

For all simulations, an experimentally measured population AIF was utilized for *C_p_*(t) [35], and the simulated time was 11 minutes. Simulations were run forward in order to generate a time dependent distribution of contrast agent within the elements. A fast exchange limit was assumed in these simulations, and hence the total voxel *R_1_* was calculated as the weighted sum of the individual tissue compartment *R*_1_ values (EES, *v_p_*, and EIS). The EES *R_1_* was calculated from the dynamic elemental CA concentrations obtained from the simulations. Specifically, for each element (which represent the EES), at each time point (*k*), the elemental concentration was converted to *R_1,elm_ via* [26]:
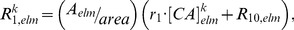
(11)where *R_10,elm_* is the baseline relaxation rate of the elemental tissue, [*CA*]*^k^_elm_* is the concentration of the contrast agent in the element at time point *k*, *r_1_* is the contrast agent-specific relaxivity (in units of mM^−1^s^−1^), *A_elm_* is the elemental area, and area is the total voxel area. For the simulations presented here, *r_1_* was set to be 4.7 mM^−1^s^−1^ (appropriate for 7T, [35]) and *R_10elm_* was 0.5 s^−1^. The ratio of *A_elm_*/area is introduced so that the contribution of each element to the total voxel *R_1_* is weighted based on the area of the element relative to the whole domain; that is, 2.0×10^−6^ mm^2^/0.0625 mm^2^, or 3.2×10^−5^. The vascular component of the voxel *R_1_* will also change with time, and thus requires a similar dynamic calculation:
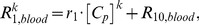
(12)where *C_p_* is defined by a population AIF as above, *R_10,blood_* is set to 0.5 s^−1^, and *r_1_* remained the same as before. In this case, it was not necessary to weight *R_1,blood_* by a subdivided area since the contrast agent is assumed to be uniformly distributed within the vessel; instead, the area weighting of the entire *v_p_* region is handled in Eq. (13). The total voxel *R_1_* is then simply the weighted sum of the *R_1_* contribution from each of the three tissue fractions: the EES, the vascular space, and the EIS. Note that the EIS *R_1_* does not change with time as contrast agent is assumed not to enter the cells; thus *R_1,eis_* was the same for each time step and was set to 0.5 s^−1^. The composite voxel *R_1_* was calculated by:
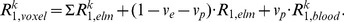
(13)


The total voxel SI at each time step *k* could then be calculated utilizing the standard gradient echo equation:
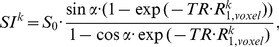
(14)where we have assumed the echo time (*TE*) is much less than the apparent transverse relaxation time (T_2_*), *TR* is the repetition time for the scan, *α* is the flip angle (25°), and *S_0_* is the baseline signal intensity. For simplicity, we set *S_0_*  =  1 for all calculations. Additionally, the repetition time was selected to be 5 ms for all calculations.

Performing this calculation for each time step, *k*, over the simulation resulted in a dynamic *SI* for the whole voxel. Simulations were run for various *D* ranging from 3×10^−5^ to 3×10^−3^ mm^2^/s. These values were chosen to give a sufficient range of diffusion coefficients around in vivo measurements of gadolinium diffusivity, which has been reported as a mean *D* of 2.08 × 10^−4^ mm^2^/s (range: 1–3.4×10^−4^ mm^2^/s) [36]. This specific range of *D* was evaluated for gadoterate meglumine (Gd-DOTA). A similar *D* value was calculated for gadopentetate dimeglumine (Gd-DTPA, 2.6×10^−4^ mm^2^/s) [37], which has a comparable molecular weight. It is noted that other Gd chelates may have a different range of average *D* values; however, the range evaluated in this work covers two orders of magnitude and hence encompasses typical *D* values for a variety of gadolinium based-contrast agents. The permeability coefficient, *P*, was also varied, to achieve the aforementioned *K^trans^* values of 0.1, 0.4, and 0.7 min^−1^.

Assessing the effect of temporal resolution on the parameterization error was accomplished by means of sampling the dynamic voxel *SI* data at the different intervals. We selected temporal resolutions covering a range of those commonly encountered in preclinical and clinical studies: 1.6, 3.2, 6.4, 12.8, and 25.6 s. The resulting *SI* data sets were then fit using the extended Tofts model (2) to extract *K^trans^*, *v_e_*, and *v_p_*. The fitting procedure consisted of fitting the *SI* data using the standard conversion procedure (standard gradient echo equation coupled with the fast exchange limit assumption) and utilizing a non-linear least squares approach, with initial guesses of [0.1 min^−1^, 0.1, 0.01] for *K^trans^*, *v_e_*, and *v_p_*, respectively. These values could then be directly compared to *K^trans^* assigned during the simulation, and the *v_e_* and *v_p_* values defined by the domain to assess parameterization error due to diffusion and temporal resolution. The parameterization error was calculated as: 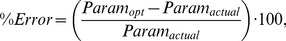
(15)where *Param_opt_* is the optimized parameter value obtained from fitting the extended model (i.e., Eq. (2)) to the data and *Param_actual_* is the parameter value assigned in the simulation.

## Results

**Figure 2:**
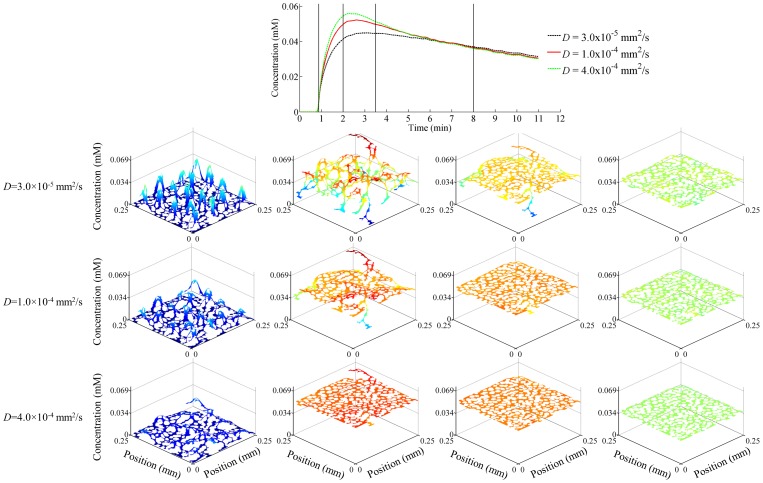
Voxel EES Contrast Agent Concentration Distribution as a Function of the Coefficient of Diffusion. The concentration distributions and overall voxel EES concentration are shown for the central voxel of interest for the simulation with *v_e_*  =  0.39, *v_p_*  =  0.03, and *K^trans^*  =  0.4 min^−1^. The top figure shows the overall voxel EES concentration for three representative coefficients of diffusion (*D*). The lower panels demonstrate spatial voxel contrast agent concentration distribution as a function of diffusion (increasing from top to bottom) and time (increasing from left to right). The four time points are indicated by the black vertical lines on the voxel concentration plot. Note that at a high *D* value (4×10^−4^ mm^2^/s), the contrast agent concentration is nearly uniform throughout the domain at each time point. However, at lower (and more physiologically relevant) *D*, there is substantial heterogeneity within the domain, especially during the early times points during which the vascular concentration is highly dynamic. Additionally, it is important to note that although the peaks become less distinguished with increasing *D* at the earliest time point, the overall domain EES CA concentration increases with increasing *D*. This is due to the fact that with increasing *D*, the CA is able to diffuse away from the vessel periphery and into the domain. Specifically, the median concentration in the *v_e_* domain space is 7×10^−4^ mM, 0.0026 mM, and 0.0047 mM for each *D* value shown (from lowest to highest).

**Figure 3:**
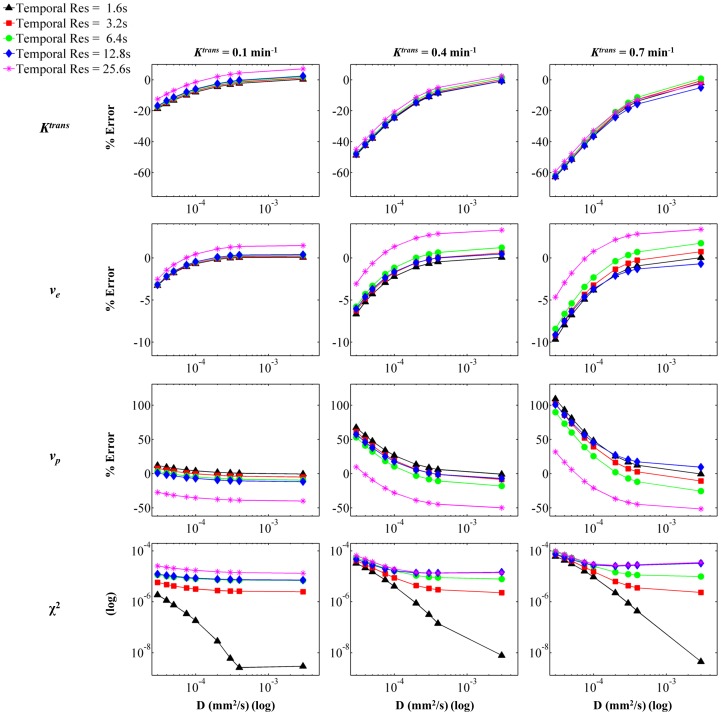
Parameterization Error as a Function of Contrast Agent Diffusion, Temporal Resolution, and *K^trans^*. Voxel *SI* was calculated from each concentration distribution for the appropriate combination of parameters, and *SI* curves were generated using a range of temporal resolutions. The resulting curves were fit using the extended model. The panels show percent error from input value for (from top to bottom) *K^trans^*, *v_e_*, and *v_p_*, and goodness of fit as measured by χ^2^. The columns indicate input *K^trans^* value, increasing from left to right. The simulation utilized *v_e_*  =  0.39 and *v_p_*  =  0.03.

**Figure 4:**
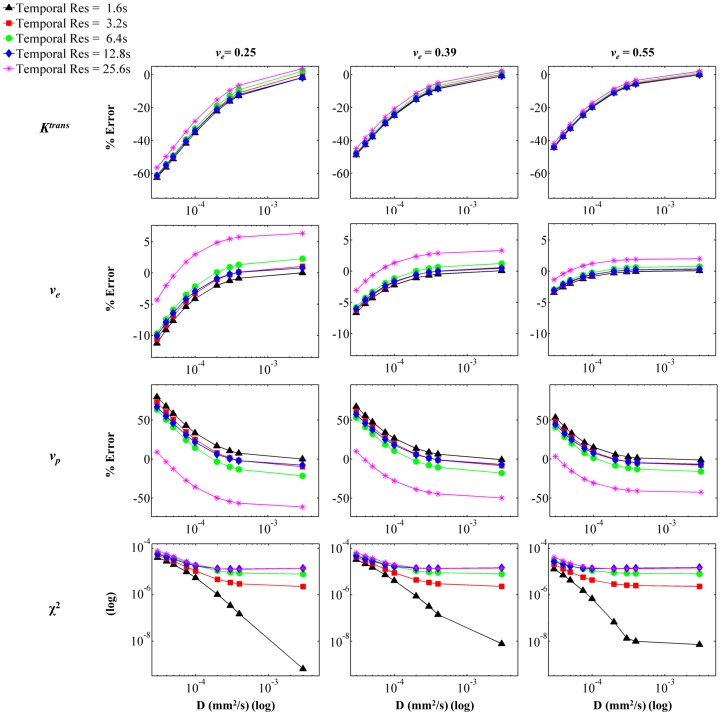
Parameterization Error as a Function of Contrast Agent Diffusion, Temporal Resolution, and *v_e_*. Voxel *SI* was calculated from each concentration distribution for the appropriate combination of parameters, and *SI* curves were generated using a range of temporal resolutions. The resulting curves were fit using the extended model. The panels show percent error from input value for (from top to bottom) *K^trans^*, *v_e_*, and *v_p_*, and goodness of fit as measured by χ^2^. The columns indicate input *v_e_* value, increasing from left to right. The simulation utilized *K^trans^*  =  0.4 and *v_p_*  =  0.03.

**Figure 5:**
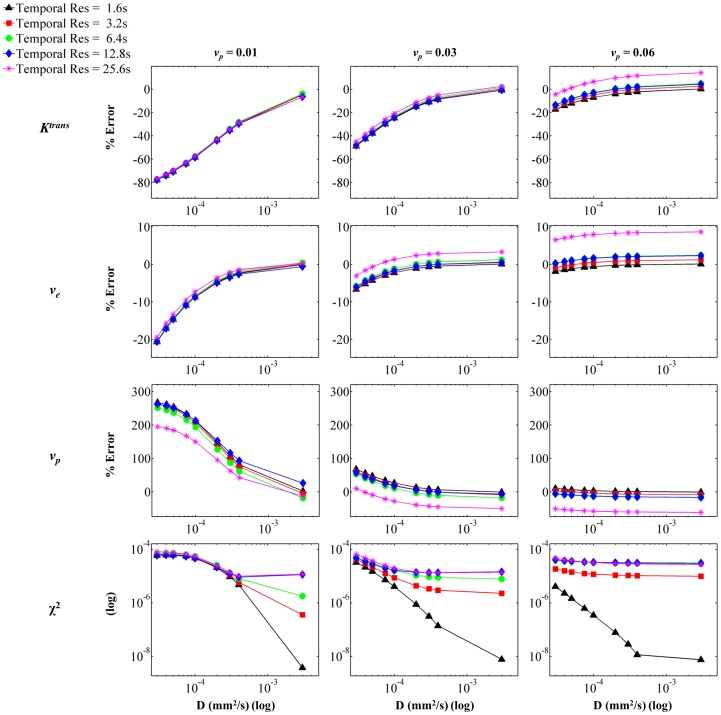
Parameterization Error as a Function of Contrast Agent Diffusion, Temporal Resolution, and *v_p_*. Voxel *SI* was calculated from each concentration distribution for the appropriate combination of parameters, and *SI* curves were generated using a range of temporal resolutions. The resulting curves were fit using the extended model. The panels show percent error from input value for (from top to bottom) *K^trans^*, *v_e_*, and *v_p_*, and goodness of fit as measured by χ^2^. The columns indicate input *v_p_* value, increasing from left to right. The simulation utilized *K^trans^*  =  0.4 and *v_e_*  =  0.39.

The results of the simulations are shown in Figs. 2, 3, 4, and 5 below. Fig. 2 shows a representative example of the domain EES contrast agent concentration and the spatial domain EES contrast agent distribution as a function of coefficient of diffusion at various times along the dynamic acquisition. The results are shown for *K^trans^* of 0.4 min^−1^, *v_e_* of 0.39, and *v_p_* of 0.03. The top graph in the figure shows the overall voxel EES contrast agent concentration (i.e., the sum of the amount of contrast agent in each element divided by the total *v_e_* area) as a function of time for three representative coefficients of diffusion utilized in the simulations. The panels in the figure illustrate the spatial variation of the contrast agent concentration within the voxel EES as a function of diffusion coefficient, with *D* increasing from top row to bottom row, and time increasing from left to right. The time represented by each of the four panels is indicated by the vertical black lines in the top voxel concentration plot; specifically, 0.88 minutes (the time of the peak of the AIF utilized in the simulations), 2 minutes, 3.5 minutes, and 8 minutes. These times were chosen to sample the early, dynamic period of the wash in and wash out, as well as a later time point when the contrast agent is more homogeneously distributed. The figure illustrates the effect of diffusion on both the total voxel CA concentration, as well as the distribution of the CA in the voxel domain over time. At early time points, when the difference between the CA concentration in the vasculature and that in the domain is high, CA moves into the domain in the regions at the vessel boundaries, as indicated by the distinct peaks in the left column of panels. In the case of a low *D* (top left panel), the CA remains proximal to the vasculature. However, as *D* increases, the CA disperses into the domain. This behavior is observed by comparing the panels in the left column, wherein the peaks surrounding the vessels become less distinct as *D* increases. Note, though, that while the peaks decrease, the total amount of CA in the domain EES increases with increasing *D*; this can be seen in the concentration curves shown in the top panel. For example, for the earliest time point indicated, the median CA concentration in the EES is 7×10^−4^ mM for *D*  =  3×10^−5^ mm^2^/s, 0.0026 mM for *D*  =  1×10^−4^ mm^2^/s, and 0.0047 mM for *D* = 3×10^−4^ mm^2^/s. Thus, with increasing *D* the CA traverses further from the vascular periphery, which allows more CA to move into the domain while also increasing the domain homogeneity.

Figs. 3, 4, and 5 show the results for parameterization error. The simulations were run with various domains in order to vary *v_e_* and *v_p_*. Additionally, the input value of *K^trans^* and *D* were varied, as was the temporal resolution. Each figure shows the result of parameterization error as it depends on *D* and temporal resolution. In all figures, the rows represent (from top to bottom) parameterization error for *K^trans^*, *v_e_*, and *v_p_*, and χ^2^ (to measure goodness of fit). Additionally, *D* is indicated by the *x*-axis of each panel (log scale), and the temporal resolution is indicated by the five different curves on each panel. Fig. 3 shows the effect of *K^trans^*, which increases in each column from left to right. Similarly, Fig. 4 demonstrates the effect of *v_e_* while Fig. 5 shows the effect of *v_p_*. In all cases, the parameterization error trends toward 0 for the highest temporal resolution with increasing *D*. This corresponds with the assumption of instantaneous CA distribution in the domain; as *D* increases and the instantaneous distribution is approached, the accuracy with which the extended model is able to represent the simulated data increases. Temporal resolution demonstrates a strong effect on parameterization error in all three figures. In general, decreasing temporal resolution introduces error in parameterization due to the inability to detect highly dynamic features of the curve, such as the wash-in features and the quick peak concentration in the vasculature. Thus, in most cases, decreasing temporal resolution causes an increase in parameterization error.

The extended model parameterization errors for *K^trans^* ranged from –58% to 12% over the range of *D* expected for gadolinium chelates in tissue (i.e., approximately 1×10^−4^ to 4×10^−4^ mm^2^/s), temporal resolutions, *K^trans^*, *v_e_*, and *v_p_* evaluated. With increasing *K^trans^*, the parameterization error for all three parameters increases. This is due to the fact that *K^trans^* reflects permeability in our model, and an increase in *K^trans^* results in an increased amount of CA moving into the system at the periphery of the vessels, thus increasing the disparity between concentration at the vessel periphery and concentration in the further regions. The magnitude of the errors associated with the parameterization of *v_e_* is relatively small when compared to the errors in *K^trans^*; for *v_e_* the errors ranged from −9% to 8% over the same range of *D*. Increasing *v_e_* causes a slight decrease in error across the three parameters. This can be attributed to the fact that increasing *v_e_* decreases the barriers to CA diffusion (i.e. decreases the number of cells), thus allowing the CA to more rapidly approach a homogenous distribution within the domain. Finally, errors for *v_p_* in the appropriate range of *D* encompassed −60% to 213%. Increasing *v_p_* causes a distinct decrease in parameterization error for *K^trans^*, *v_e_*, and *v_p_*. As *v_p_* increases, the vessel density increases, thus decreasing the distance between any given tissue region and its nearest source of perfusion. Consequently, increasing *v_p_* serves to decrease the heterogeneity of the CA distribution and subsequently decreases parameterization error.

When considering the average diffusion coefficient value measured for gadolinium chelates (approximately 2×10^−4^ mm^2^/s), the errors for *K^trans^* ranged from −44% to 10%, the errors for *v_e_* ranged from −5% to 8%, and the errors for *v_p_* ranged from −60% to 152% over the implemented *K^trans^*, *v_e_*, and *v_p_* values and over the range of temporal resolutions investigated. Specifically, for the highest temporal resolution (1.6 s) the errors for *K^trans^* ranged from −44% to −4%, *v_e_* errors ranged from −4.8% to −0.2% and errors for *v_p_* ranged from 1.6% to 146%. For all three parameters, the largest parameterization error is noted in the simulation domain with a *v_p_* value of 0.01, and the error is quickly minimized with increasing *v_p_*. This again reiterates the large impact of *v_p_* on CA delivery, which ultimately also affects domain CA homogeneity and hence parameterization error.

The quality of fit of the extended model for each simulated voxel SI is quantified by χ^2^, which is shown in the bottom row of panels in Figs. 3, 4, and 5. The χ^2^ values for the simulations ranged from 3.28×10^−5^ to 2.61×10^−9^, and in all figures the χ^2^ values are plotted on a log scale in order to emphasize differences between temporal resolutions. In each simulation, and for all temporal resolutions, the magnitude of χ^2^ decreases with increasing *D*, indicating that as *D* increases, the extended model is better able to fit the simulation data. This is expected, as increasing *D* reduces the heterogeneity in the domain and results in a more ‘well-mixed’ voxel, as is assumed in the extended model. Additionally, in most cases the value of χ^2^ indicates a better fit with increasing temporal resolution. This is again expected, as the increased temporal resolution allows for identification of salient dynamic features of the SI curve, such as the rapid uptake.

## Discussion

The simulations utilized for this work aim to evaluate the effect of intra-voxel contrast agent diffusion on measured voxel signal intensity time courses, which will subsequently affect the ability of the extended Tofts model to estimate the pharmacokinetic parameters characterizing the voxel. These simulations consider physically realistic voxels, with cell and vessel distributions included *via v_e_* and *v_p_*. Additionally, realistic temporal resolutions are utilized to most closely approximate how dynamic data would be acquired in a typical *in vivo* experiment. The results of the simulations indicate that diffusion plays a large role in the distribution of contrast agent within the domain and hence on the total domain *SI*. Notably, the maximum concentration of contrast agent within the domain EES is affected by the distribution of contrast agent within the domain, and this distribution is determined by diffusion. This can be seen in the top plot of Fig. 2, where the shape and maximum voxel concentration are dependent on diffusion. Specifically, there is a noticeable difference in the slope of the wash-in portion of the curves, which is dependent not only on *D* but also on the *K^trans^* and the voxel *v_p_*. In considering the concentration distributions in the lower panels of Fig. 2, it is apparent that *D* has a distinct effect on the amount of contrast agent in the system as well as the distribution of the contrast agent within the voxel. In particular, the distributions in the first column display noticeable peaks of concentration around the vessels, as would be expected. However, these peaks are exaggerated in the lower *D* case, as the contrast agent does not diffuse away into the domain. As *D* increases, and the contrast agent diffuses further away from the vessels, the concentration within the overall domain becomes larger, and the peaks around the vessels are reduced. In all cases, however, the contrast agent eventually distributes within the total voxel domain; this is indicated by the curves converging during the later time points as well as the more uniform appearance of the last column of contrast agent concentration distribution plots.

The system utilized in this work incorporates a parameter *P* that dictates the permeability of the vessel at the boundary. Eq. (2) assumes instantaneous mixing and hence homogeneous distribution; thus, we opted to assign *P* so that at high *D* (i.e. *D*>10^−3^ mm^2^/s, when the contrast agent had sufficient mobility to distribute uniformly within the domain) the amount of contrast agent in the domain was the same as defined by the standard approach for a given *K^trans^*. Given this stipulation, it should be noted that even at higher *D* values, when the amount of contrast agent in the system is nearing that predicted by the Tofts model, the distribution of the contrast agent, and thus the voxel *SI*, will still vary with *D* until *D* is sufficiently high enough to provide an instantaneously homogeneous distribution throughout the voxel. This also means that, at lower *D*, the amount of contrast agent in the system would be less than that predicted by the standard approach. We believe that this is an appropriate, and physically relevant, approach to the system since the amount of contrast agent entering the EES will be driven by the gradient at the perimeter of the vessel, and hence if the contrast agent is not diffusing away from the vessel, then the gradient will be reduced and less contrast agent will move into the EES. However, we note that at the highest *D* value presented in the figures (3×10^−3^ mm^2^/s – more than an order of magnitude higher than the *D* expected for the common gadolinium chelates in tissue), the parameterization error at the highest temporal resolution is trending toward (and quite nearly) 0 in all cases.

The results for the percent parameterization error for the various voxel configurations are shown in Figs. 3, 4, and 5. The results indicate that, for values in the range of that expected for gadolinium in tissue, diffusion does indeed have a substantial effect on the parameterization error. For all parameters, the error plateaus as the coefficient of diffusion increases. Additionally, in most cases, the error is reduced as the magnitude of the temporal resolution is reduced. However, it is interesting to note that in some cases, the larger temporal resolutions actually result in a smaller absolute error, or they result in an overestimation of the parameter. This is likely due to the use of a least-squares regime for curve fitting as well as the fact that the various temporal resolutions affect the features of the *SI* curve that are retained, and hence can significantly alter the fit. For example, in most cases the extended model overestimates *v_p_*, but for some larger temporal resolutions the model underestimates *v_p_*. This can be attributed to the fact that the initial steep increase in the contrast agent concentration in both the plasma and the tissue is not sampled due to the longer temporal resolution.

Generally speaking, the magnitude of the errors associated with the parameterization of *v_e_* is relatively small when compared to the errors in *K^trans^* and *v_p_*. This is due to the fact that the assignment of *v_e_* is driven by the shape of the latter portion of the *SI* curve during which the plasma concentration, and hence the entire voxel, is less dynamic. The effect of diffusion is mainly perceived in the earlier portions of the curve, when the system is highly dynamic and the movement of the contrast agent into and out of the voxel is rapid. Diffusion plays a significant role in the larger errors associated with *K^trans^* and *v_p_*. In a low *D* situation, the contrast agent will initially extravasate into the system for a brief period of time, but the amount of contrast agent that enters the tissue is limited as the contrast agent does not move away from the blood vessel boundaries. Hence, there is a brief, quick increase in the signal intensity caused by movement of contrast agent into the tissue. This rapid uptake coupled with the plasma contribution to the total voxel *SI* is likely what causes the large error of *v_p_*. However, because the rapid extravasation of the contrast agent does not continue, and the slope of the total voxel *SI* curve flattens, the optimized *K^trans^* is underestimated. Thus, in most cases investigated, *v_p_* is overestimated in order to account for the increase in *SI* for which *K^trans^* cannot account due to the short duration of the said increase.

Overall, the effect of intra-voxel diffusion on parameterization error is emphasized by increasing *K^trans^* and decreasing *v_p_*. In Figs. 3 and 5, the range of percent error for each parameter increases with increasing *K^trans^* and decreasing *v_p_*. This would be expected since, physiologically, these two parameters affect the introduction of the contrast agent to the EES. As *K^trans^* increases, which relates to permeability in our simulations, standard analysis methods (which assume a well-mixed EES) expect the amount of contrast agent that enters the system to increase. However, since *D* affects the amount of contrast agent which can move into the EES, the *SI* does not match that predicted by the standard analysis. Thus, the parameterization errors increase with increasing *K^trans^* due to the effect of *D* on contrast agent amount and distribution. For similar reasons, as *v_p_* decreases, and thus the boundaries at which the contrast agent can enter the EES decrease, absolute parameterization errors increase. It is important to note that for well-vascularized tissue (i.e., higher *v_p_*), and moderate temporal resolutions the parameterization errors are relatively small. Specifically, for the high *v_p_* domain evaluated here (for *K^trans^*  =  0.4 min^−1^, *v_e_*  =  0.39 and *v_p_*  =  0.06), and a temporal resolution of 6.4 sec, the parameterization errors over the range of *D* values for gadolinium chelates in tissue for *K^trans^*, *v_e_*, and *v_p_* are −2.8% to 2.3%, 1.7% to 2.2%, and −15% to −12.5%, respectively. These errors are low, and result in *K^trans^* values ranging from 0.387 to 0.407 min^−1^, *v_e_* from 0.397 to 0.399, and *v_p_* from 0.051 to 0.053. Thus, in well-vascularized regions, the effect of intra-voxel diffusion is minimized.

The results shown here are presented for a typical voxel size utilized for preclinical evaluations (250 µm^2^). A clinically relevant voxel size would be significantly larger (i.e. on the order of 1 mm^2^). Unfortunately, the size of the domain required to simulate a clinically-sized voxel is computationally restrictive. However, the results presented here can be utilized in consideration of a larger domain. Namely, it would be expected that *v_p_* and *K^trans^* will have a large influence on the effect of diffusion since they affect contrast agent delivery to the EES. An additional consideration is the location of the vasculature in the domain. If the vasculature is spaced across the voxel domain, this will have a different effect than if the vasculature is clustered in a particular region of the voxel. In the simulations presented here, the vasculature was spaced throughout the domain. However, clustering of the vasculature would presumably affect the simulations, as this would generate regions of the domain which were not perfused and hence longer diffusive distances which the contrast agent must traverse to equilibrate within the domain. This would certainly be of increased consideration in a larger domain.

A notable limitation of this model is that we assume a constant, replenished concentration of contrast agent in the vasculature that is equal to the measured arterial input function from preclinical studies. Thus, this work did not account for a varying concentration in the vasculature beyond the dynamically evaluated *C_p_*(*t*). Specifically, flow is not considered in this model, and hence *K^trans^* in this work represents the permeability-surface area (PS) product (i.e., the permeability-limited case). Thus, the results presented may be most applicable in (for example) the brain where pathological conditions affect the permeability of the normal blood brain barrier in such a way that the permeability-limited assumption is valid [4], [38]. Future efforts will aim to explore the flow-limited case in which a new definition for *K^trans^* at the boundary of the vessel would be required. Secondly, we assume a *S_0_* value of 1 for the entire area of the voxel. This value was utilized to simplify the calculations of the total voxel domain in regard to the three tissue components. However, this value would actually depend on tissue characteristics such as proton density, and would likely not be uniform across the various components.

The work presented here highlights the potential issues associated with the typical quantitative approach which ignores diffusion in DCE-MRI analysis. Through simulations, we have shown that *D* in the range of that expected for gadolinium in tissue may have a significant effect on the voxel *SI* and hence may be important to consider in quantitative analysis. However, this paper does not serve to present a solution to the issue. Additionally, in regards to intra-voxel versus inter-voxel diffusion, it will likely be quantitatively difficult to discern between the effects of the two. In considering a possible solution, a potential approach would be an inversion problem where the voxel-wise diffusion coefficient could be quantified; this diffusion value would account for both inter- and intra-voxel diffusion. Unfortunately, the distribution of the contrast agent within the voxel is a very difficult problem to characterize and may not be reasonably quantifiable. However, the voxel-wise assignment of *D* is a problem which is manageable using current inversion techniques, and is a current topic of interest in our laboratory.

## Conclusion

The purpose of this work was to investigate the effect of intra-voxel diffusion on quantitative DCE-MRI analysis. Utilizing a FEM of a physiologically relevant voxel domain and assigning *D*, *K^trans^*, *v_e_*, and *v_p_*, we were able to quantify the effect of diffusion and temporal resolution on voxel *SI*. These simulations also allowed for quantification of the parameterization error attributed to analysis *via* the standard and extended models. The results show that both diffusion and temporal resolution have a substantial effect on parameterization error, hence indicating that it may not be reasonable to ignore the effect of diffusion in quantitative analysis of DCE-MRI data. Future efforts will focus on methodologies to account for this phenomenon in the analysis of DCE-MRI data.

## Acknowledgments

We thank Drs. Jared Weis, Ph.D. and Michael Miga, Ph.D., for many informative discussions. Additionally, we thank Mr. Nateneal Semmineh for his help with the packed domains. We thank the National Cancer Institute for funding through R01CA138599, R01CA158079, R25CA092043, P30CA68485, P50CA098131, and U01CA174706.
